# Advances in liquid biopsy approaches for early detection and monitoring of cancer

**DOI:** 10.1186/s13073-018-0533-6

**Published:** 2018-03-20

**Authors:** Anna Babayan, Klaus Pantel

**Affiliations:** Department of Tumor Biology, University Medical Center Hamburg-Eppendorf, Martinistraße 52, 20246 Hamburg, Germany

**Keywords:** Liquid biopsy, Circulating tumor cells, ctDNA, Minimal residual disease, Early detection, Biomarker

## Abstract

Progress in sensitive analytical approaches has opened new avenues for the detection of cells or products such as circulating cell-free DNA released by tumors. These ‘liquid biopsies’ are being explored in clinical trials for early cancer detection, prediction of recurrent disease, and assessment of therapeutic resistance mechanisms.

## Liquid biopsy approaches in cancer

The analysis of tumor cells and tumor-derived products that are detectable in the blood and other body fluids was introduced by Pantel and Alix-Panabieres as ‘liquid biopsy’ [1], and has attracted substantial interest in recent years. Besides circulating tumor cells (CTCs), circulating cell-free tumor DNA (ctDNA) is the second most investigated analyte of liquid biopsies [2]. More recently investigated liquid biopsy analytes include circulating microRNAs and long non-coding RNAs, tumor-derived exosomes, and tumor-educated platelets [2].

Clinical applications of liquid biopsies in early-stage cancer patients include early detection of small tumors, improved risk assessment, and monitoring of minimal residual disease (MRD) [2]. This diagnostic information can now be implemented into new clinical trials designed to cure cancer patients before overt metastasis. Many liquid biopsy tests have been established and validated over the past 10 years [2], and some have already reached the clinic (for example, liquid-based oncotypeIQ® tests and the FDA-approved cobas® EGFR mutation test v2 and CellSearch®-based CTC enumeration). However, the majority of the new tests suffer from insufficient clinical and technical validation and lack clinical utility [3, 4].

Liquid biopsy approaches have the potential to become a cornerstone of personalized medicine if these challenges can be addressed and improvements in sensitivity can be achieved. Recent ctDNA and CTC tests have provided promising results for early cancer detection, MRD detection, and prediction of disease recurrence [2, 5–9]. Here, we focus on recent approaches for early detection and monitoring of cancer using ctDNA.

## Early detection of cancer

Early detection of cancer through a screening program for healthy and high-risk individuals is a key application of liquid biopsy approaches. Current therapeutic strategies allow the successful treatment of many patients if the disease is detected early enough, whereas metastatic disease still remains incurable with very few exceptions (for example, small liver metastases in colon cancer).

The choice of biomarkers for early detection is crucial. Biomarkers that can be detected and validated in cancer patients with advanced disease may lack specificity and sensitivity for early detection, for example, carcinoembryonic antigen (CEA), which is currently used to monitor colorectal carcinoma treatment, and can also be present at raised levels in gastric, pancreatic, lung, and breast cancer, as well as in some non-neoplastic conditions. Such markers are usually present at lower concentrations in early cancer stages compared to late stages, and the biology of these two disease states varies, so a late stage marker might not be suitable to detect small tumors in early stages. Blood-based markers of early lesions might also be masked by other comorbidities such as chronic inflammatory diseases, as well as by the accumulation of cancer-related mutations with age in healthy individuals [2].

These limitations are illustrated by the recent work of Cohen et al. [5] who introduced the CancerSeek panel for detection of the eight most common cancers. This complex approach combined the evaluation of eight soluble tumor biomarkers (including standard tumor markers such as CEA) with ctDNA analysis of cancer-related mutations in 16 genes. The panel reached an overall median sensitivity of 70% with specificity at ≥ 99%, but significant differences in sensitivities were observed among the tumor types analyzed (for example, 98% in ovarian cancer, 60% in lung cancer, and 33% in breast cancer) [5]. Moreover, the authors analyzed only healthy controls; thus, the high specificity of the CancerSeek approach requires further validation with a non-cancer control with comorbidities such as inflammatory diseases that are common in older individuals.

## Monitoring of minimal residual disease in early-stage cancer patients

Liquid biopsy tests for monitoring of MRD in early-stage cancer patients face similar challenges to other tests for early detection because of the low concentration of ctDNA (and other liquid biomarker analytes) in the blood [2]. Tie et al. [6] demonstrated the ability of ctDNA analysis of blood samples, obtained from stage II colon cancer patients after surgical removal of the primary tumor, to predict recurrence at 36 months with 48% sensitivity and 100% specificity. In the study by Abbosh et al. [7] detection of ctDNA mutations that were also present in the respective primary tumor was predictive for relapse in 93% of cases with a median of 70 days prior to radiologic confirmation. The authors estimated the costs for the patient-tailored tests at US$1750 per patient, which might be too high for the routine implementation of the approach as a cancer monitoring tool. Both studies demonstrate the feasibility and potential clinical value of ctDNA analysis for MRD monitoring. However, ctDNA detection required knowledge of primary tumor-specific mutations and the mutational spectrum may change during progression from MRD to overt metastatic disease.

ctDNA analysis without prior knowledge of the primary tumor genetics was applied in a recent study by Chaudhuri et al. [8] of stage I–III lung cancer patients. By utilizing the highly sensitive approach known as cancer personalized profiling by deep sequencing (CAPP-Seq) to target 128 genes, these authors were able to show that the detection of ctDNA following the initial treatment of the primary tumor could predict progression in 72% of patients an average of 5.2 months prior to radiologic evidence. Remarkably, ctDNA was already detectable in 94% of patients experiencing recurrence at the ‘MRD landmark’ time point, the first post-treatment blood draw within 4 months of treatment completion [8].

Goh et al. [9] identified chromosome 1q23.1 amplification as enriched in tumor-initiating cells in breast cancer patients. Detection of the amplification (as the average copy-number ratio of genes *TUFT1*, *S100A7*, and *S100A8* by droplet digital PCR) in ctDNA samples at first diagnosis was predictive of relapse within 5 years in 67% of early-stage patients and within 3 years in 40% of patients with locally advanced breast cancer, with 100% specificity in both cohorts. Taken together, these results demonstrate the power of ctDNA analysis in predicting MRD in cancer patients.

## Guiding precision therapy and monitoring treatment responses

In addition to cell-free DNA (cfDNA)-based screening and early detection tests, cfDNA-guided monitoring of therapy response is being investigated. Serial cfDNA analyses during the course of treatment may allow early detection of the emergence of resistance-associated mutations. In a prospective study, Goodall et al. [10] detected mutations that reverted germline and somatic DNA repair mutations back in frame in patients treated with the poly (ADP-ribose) polymerase (PARP) inhibitor olaparib. Monitoring of these mutations, which emerge under the selective pressure of therapy and drive drug resistance, has clinical relevance for personalized therapy. Similarly, Siravegna et al. [11] performed ctDNA analysis of patients with colorectal cancer, and observed the emergence of *KRAS* mutations that are associated with disease progression during anti-epidermal growth factor receptor (anti-EGFR) therapy. Moreover, *KRAS* mutant clones declined in number during withdrawal of therapy, leading the authors to suggest that rechallenge with anti-EGFR therapy after a period of therapy withdrawal might be a useful therapeutic strategy for these patients. These findings provide a rationale for interventional clinical studies with cfDNA-driven decisions.

## Conclusions

Liquid biopsy approaches based on cfDNA or ctDNA analyses have provided new avenues for early detection of primary cancer and MRD. Although the feasibility of cfDNA analysis for early cancer detection has been demonstrated, the combination of classical biochemical cancer markers and/or imaging techniques with cfDNA-based approaches might improve the sensitivity of these tests [5]. However, our knowledge of the biology of carcinogenesis is still not comprehensive enough to allow the development of highly sensitive screening tests. The need for such highly sensitive techniques and their accompanying high costs are remaining hurdles for these applications.

The introduction of cfDNA-guided personalized therapy into clinical practice has already begun. On the basis of cfDNA, through *EGFR* T790 M mutation testing using the cobas® EGFR Mutation Test v2, non-small cell lung cancer (NSCLC) patients can be stratified for treatment with third-generation tyrosine kinase inhibitors without re-biopsy [4]. Despite the increasing evidence that sequential cfDNA analyses can be used to monitor MRD and therapy responses in cancer patients, there are no other FDA-approved cfDNA-based tests available. Independent assay validation by international research groups, such as the European CANCER-ID consortium (www.cancer-id.eu), is important for the implementation of liquid biopsy technologies into clinical trials. Numerous clinical trials of cfDNA and CTC assays are currently underway (www.clinicaltrials.gov), and the prospects for the clinical application of liquid biopsy approaches to improve cancer management will ultimately depend on achieving improved outcomes compared to the current standard of care.

## Abbreviations

CEA: Carcinoembryonic antigen
cfDNA: Cell-free DNA
CTC: Circulating tumor cell
ctDNA: Cell-free tumor DNA
EGFR: Epidermal growth factor receptor
MRD: Minimal residual disease
